# Differential item functioning of material deprivation assessment in households with or without children

**DOI:** 10.1371/journal.pone.0290112

**Published:** 2023-08-17

**Authors:** Maria Eugénia Ferrão, Marcel Toledo Vieira

**Affiliations:** 1 Faculty of Sciences, University of Beira Interior, Covilhã, Portugal; 2 Exact Sciences Institute, Federal University of Juiz de Fora, Juiz de Fora, Brazil; Xiangtan University, CHINA

## Abstract

**Background:**

Composite multidimensional indices are broadly used to measure child poverty and social exclusion. Many of such indices are based on EU-SILC data or similar large scale complex sampling surveys, with the household as unit of analysis. Indicators related to households with or without children may quantify the intended attribute differently depending on the household structure and characteristics of individuals, potentially compromising the assessment.

**Methods:**

We conducted statistical modelling and hypotheses tests using a two-parameter logistic item response model (IRM) and the likelihood-ratio test for DIF verification. Methods were applied to 2020 EU-SILC Portuguese data comprising 11,367 households representing a population of 4,099,052. Statistical analysis have allowed for the survey sampling design.

**Conclusion:**

Our findings demonstrate differential item functioning in the assessment material deprivation in households with or without children.

## Introduction

In contemporary times, child poverty and social exclusion (CPSE) are still some of the world’s major social challenges and have recently gained significant attention in economic agendas. Despite overall economic growth and progress, in many countries inequalities and disparities persist, affecting vulnerable groups such as children. As part of the 2030 SDG Agenda, many efforts at quantifying CPSE have been done [1–13]. It has been recognized that the needs and living conditions of children differ from those of adults [8, 14–16]. Despite the importance of developing a child-centered analysis [8, 15], restrictions imposed by data availability gives strength to a measurement on an annual basis of the most widely used dataset to analyze poverty and social exclusion in Europe, which the EU-SILC. In fact, the proposal “Towards an EU measure of child deprivation” [8, 15] is mentored to be “a careful and systematic analytical framework to identify an optimal set of robust items to be included in an EU child-specific MSD indicator for use by the European Commission and Member States in their regular social monitoring” [8, p. 854]. The proposal regarding child material and social deprivation is founded on the thematic deprivation modules gathered through the EU-SILC operation across Europe every five years. This implies that the infrequent measurement poses a constraint on regular social monitoring and the timely development of public policies. Additionally, the lack of child-centered data significantly influences the monitoring of children’s living conditions and, consequently, their overall well-being. Acting at the level of children is instrumental in breaking the cycle of intergenerational poverty and promoting improved living conditions for them. Such actions enable children living in disadvantaged circumstances to access opportunities for better health, education, and social integration. This, in turn, positions them more favorably compared to their peers in the short, medium, and long term, both during their childhood and as they transition into adulthood [17].

CPSE assessment is usually based on survey data having households as statistical units [8, 18–20]. In turn, household composition may affect validity and reliability of CPSE assessments, which are crucial properties for the purpose of monitoring and elaboration of public policies for children [21–23]. Policymakers need to know who are at (or at risk of) poverty to better design interventions and programmes, targeting poverty reduction in the short, medium and long-run. Not accounting for the conceptual difference and the specificity of the respective measurement may have implications in the diagnosis phase of the cycle of public policies aimed at reducing poverty (SDG1).

The methodological approach known as differential item functioning (DIF) allow for several aspects related to problems that may arise when differences between groups are artifacts related to the measurement process itself and, therefore, outside the scope of the measurement instrument. DIF describes a condition when the item response or indicator is dependent not only on the latent trait of the assessed unit (*e*.*g*. household) but also on the value of some additional group attribute. Thus, the validity of an assessment instrument composed by several items with the presence of DIF may be compromised for a given subgroup.

The aim of this study is to demonstrate the application of the Differential Item Functioning (DIF) approach within the framework of item response models to enhance the validity of assessing child poverty and social exclusion (CPSE). Specifically, such statistical DIF analyses are intended to diagnose those items showing an unexpectedly large difference of scoring between the focal group (households with children) and the reference group (households without children) when the two groups are matched on their total score of poverty and social exclusion. A two-parameter logistic (2PL) item response model is used to set scores on the same scale. Items chosen are those selected by Ferrão et al. [19] for quantifying the child exposure to material deprivation considering the 2017 European Statistics on Income and Living Conditions (EU-SILC) cross-sectional data, following two requirements: (i) the item is informative to measure the construct; and (ii) the item discriminates the child’s lesser or greater exposure to deprivation material in the home where he lives. The selection of items was made by applying the two parameters IRM, analyzing the respective item characteristic curves, and their information functions. Their proposal is anchored on a literature comprehensive review supporting the existence of at least four domains (Housing conditions, Financial capacity, Comfort goods, Housing environment) and a composite index that catches a large amount of information.

Thus, in this paper we apply DIF hypotheses tests in order to identify some of the EU-SILC items that perform differently for households with children and households without children. To the best of our knowledge, no study on CPSE conducted so far has demonstrated such statistical specificity with the suitable hypotheses test. Therefore, we contribute to overcome such literature gap.

Furthermore, the study has two main contributions to the field. Firstly, it provides a methodological proposal to reinforce the validity and reliability of CPSE assessment considering large scale complex sampling survey data; secondly, it statistically demonstrates which items or indicators commonly used for poverty assessment have DIF. We address such issues here by using statistical models applied to data from the 2020 EU-SILC Portuguese sample.

Under the General Data Protection Regulation [24], a research project was approved and the research team accredited by the Statistics Portugal (Instituto Nacional de Estatística, INE) in 2019, and by the EUROSTAT in 2020. Data access was granted by the Statistics Portugal (INE) through Protocol 929, and the variables used can be considered stable. However, a challenge arose in 2020 for EU-SILC data collection due to the COVID-19 pandemic, as statistical offices were unable to conduct face-to-face interviews (PAPI, CAPI) with households [25]. Nonetheless, evidence regarding income inequality and the risk of poverty in 2019 and 2020 [26] suggests that the methods of data collection may not have significantly impacted Portuguese EU-SILC data. Nevertheless, this topic requires further research.

Moreover, given that EU-SILC is widely accepted as the most relevant dataset used to analyze poverty and social exclusion in the EU, our proposal also aims to reinforce its potential of annually assessing CPSE. Therefore, this article contributes to the creation of a composite index that can be applied annually to household survey data to be used for monitoring and making public policy decisions that could promote the reduction of children’s exposure to poverty.

### Related literature

The composite index proposed by [19] to evaluate child exposure to household material deprivation enables monitoring children’s living conditions upon the EU Statistics on Income and Living Conditions (EU-SILC) survey. Their proposal stands on a two-parameter IRM [9, 27]. The rationale for using IRM instead of adding raw scores comes from the fact that the comparison among statistical units may not be suitable with raw scores. Statistical units, *e*.*g*. individuals or household representatives, at different levels of the assessed trait, differently answer to certain items due to the statistical properties of the questionnaire items. Using IRM simultaneously allows for the adjustment of a CPSE relative scale, provides the scoring on that scale [4, 9], and informs analysts about the statistical properties of the items, measurement error, and other statistical relevant elements [28, 29].

[19]’s findings suggest item parameters estimated for deprivation assessment in households with children have different estimates in households without children. In fact, the visualization of the item characteristic curves (ICC) and item information curves (IIC) comparing three different scenario (full sample of households; subsample of households with children; subsample of households without children) show that the assessment of the severity of child exposure to material deprivation depends on the scenario chosen. The power of item discrimination depends on the scenario as well. Such evidence imply that the assessment of child exposure to household material deprivation requires item specificity.

A recent review on measurement tools for child wellbeing [4] covers the literature between 2000 and August 2019. One of the article’s research questions is what statistical methods are used for scoring and construction of indices. Authors conclude that different methodological approaches to scoring and index construction have progressively been adopted making use of more sophisticated methods. Amongst the 186 articles reviewed, only 5.4% of them make use of “Rasch scoring and t-values” [4, pp. 119; Table 1]. In fact, the Rash model belongs to the family of IRM and is specifically statistically specified as one-parameter IRM. Three articles that are explicitly referred to have their empirical component based on such models and none of them incorporates DIF analysis.

## Materials and methods

### Data

This study is based upon cross-sectional data from the 2020 European Statistics on Income and Living Conditions (EU-SILC), which comprise the most relevant characteristics for conducting empirical evidence based research, such as data accuracy, statistical properties, and data availability, consisting of an excel source for the purposes of this paper. EU-SILC aims to collect timely and comparable cross-sectional and longitudinal data on income, poverty, social exclusion and living conditions [30].

The 2020 Portuguese sample consists of 11,367 households selected from a population of size 4,099,052. Approximately, 35% households include one or more children aged from 0 to 17 years, which belong to our focal group. The remaining 65% are the reference group. Both groups were defined as the result of a transformation of the household composition variable (HX060 in xHT20 data file).

The programming language and environment for statistical computing in use is R, specifically the package MIRT v1.32.1 [31, 32]. Selected items to illustrate the DIF approach are those included in the composite index of child exposure to household material deprivation [19]. Three distinctive aspects of the quantitative methods used in this study are as follows: (1) the family of statistical models, specifically the multidimensional item response model and the generalized linear model, allowing for the complex sampling design of the EU-SILC; (2) open source tools, including those based on R code, capable to deal with big identifiable data; (3) reliable cross-sectional and longitudinal data sourced by the Eurostat, which access for research purposes was allowed under the accreditation of the host organization by the Eurostat in 2020.

Table 1 presents items included in the composite index and their coding from primary data [33].

**Table 1:** EU-SILC 2020 questionnaire items selected and coding.

Variable	Domain	Label	Categories	Item coding
HX120	Housing conditions	Overcrowded household	0:No 1:Yes	I1(overcrowded house) = 0, No = 1, Yes
HS160	Housing conditions	Problems with the dwelling: too dark, not enough light	1:Yes 2:No	I2 (Dimly lit dwelling) = 0, HS160 = 2 = 1, otherwise
HH050	Financial capacity	Ability to keep home adequately warm Domain/Area	1:Yes 2:No	I3 (Home adequately warm) = 0, HH050 = 1 = 1, otherwise
HS011	Financial capacity	Arrears on mortgage or rental payments [Whether the household has been in arrears on mortgage or rental payments in the past 12 months]	1:Yes, once 2:Yes, twice or more 3:No	I4 (Arrears on mortgage or rent payments) = 0, HS011 = 3 = 1, otherwise
HS011_f		FLAG	-2: Not applicable -1: Missing data 1:Non-missing data	I4 = 0, HS011_f = -2
HS021	Financial capacity	Arrears on utility bills [Whether the household has been in arrears on utility bills in past 12 months]	1: Yes, once 2: Yes, twice or more 3: No	I5 (Arrears on utility bills) = 0, HS021 = 3 = 1, otherwise
HS021_f		FLAG	-2: Not applicable -1: Missing data 1:Non-missing data	I5 = 0, HS021_f = -2
HS031	Financial capacity	Arrears on hire purchase installments or other loan payments [Whether the household has been in arrears on hire purchase instalments or other loan payments (non housing-related debts) in past 12 months]	1: Yes, once 2: Yes, twice or more 3: No	I6 (Arrears on hire installments or other loan payments) = 0, HS031 = 3 = 1, otherwise
HS031_f		FLAG	-2: Not applicable -1: Missing data 1:Non-missing data	I6 = 0, HS031_f = -2
HS050	Financial capacity	Capacity to afford a meal with meat, chicken, fish (or vegetarian equivalent) every second day	1: Yes 2: No	I7 (Capacity to afford a meal…) = 0, HS050 = 1 = 1, HS050 = 2
HS090	Comfort goods	Do you have a computer?	1: Yes 2: No–cannot afford 3: No–other reason	I8 (Computer) = 0, HS090 = 1,3 = 1, HS090 = 2
HS110	Comfort goods	Do you have a car?	1:Yes 2: No–cannot afford 3: No–other reason	I9 (Car) = 0, HS110 = 1,3 = 1, HS110 = 2
HS170	Housing environment	Noise from neighbours or from the street [Noise from neighbours or noise from the street (traffic, business, factories, etc.]	1: Yes 2: No	I10 (Noise) = 0, HS170 = 2 = 1, HS170 = 1
HS180	Housing environment	Pollution, grime or other environment problems [Pollution, grime or other environmental problems in area caused by traffic or industry]	1: Yes 2: No	I11 (Pollution) = 0, HS180 = 2 = 1, HS180 = 1
HS190	Housing environment	Crime, violence or vandalism in the area	1: Yes 2: No	I12 (Crime,violence) = 0, HS190 = 2 = 1, HS190 = 1
HX060		Household familiar composition		I13 (DIF variable) = 0, without children = 1, with children

Source: Statistics Portugal 2021 EU-SILC Portuguese sample under Protocols number 929, RPP 380/2020-EU-SILC (EUROSTAT); Household Data (H-file)

Table 2 presents relative frequencies per type of group computed with the cross-sectional weights variable (DB090 in xDT20 file). For example, within the focal group 12.24% of households are overcrowded (I1), 12.80% have no capacity to keep home adequately warm, and so on.

**Table 2:** Empirical distribution conditional on group.

Item (%)												
Group	I1	I2	I3	I4	I5	I6	I7	I8	I9	I10	I11	I12
Reference	2.07	6.94	22.95	1.38	2.66	1.01	3.48	8.04	6.47	24.81	13.50	6.53
Focal	12.24	6.40	12.80	3.68	4.23	1.79	2.11	3.94	3.42	25.55	12.80	7.00
Total	5.61	6.75	19.41	2.18	3.20	1.28	3.00	6.61	5.41	25.07	13.26	6.70

Source: Own calculation upon 2020 EU-SILC Portuguese sample, weights considered.

## Methods

A two-parameter logistic (2PL) item response model is used as scoring method, following recent papers [9, 19], for which the probability of a certain item to be equal to 1, *i*.*e*. *Y*_*i*_ *= 1*, conditional on *θ* is posited by

$$P\left(Y_{i}=1\;|\;\theta \right)=\frac{exp\left[D\alpha_{i}\left(\theta -\beta_{i}\right)\right]}{1+exp\left[D\alpha_{i}\left(\theta -\beta_{i}\right)\right]}$$ (1)

where *α*_*i*_ corresponds to the discriminating power of the item *i*, *β*_*i*_ corresponds to the severity of CPSE caught by the item *i*. D is a constant equal to 1.7, and serves as scaling the logistic link function to be close to the normal ogive link function. The probability function (1) describes an S-shaped curve that specifies the relationship between the probability of being exposed to a certain item deprivation, *Y*_*i*_ = 1, given the level of CPSE (*θ*) experienced by the child, and the item parameters (*α*_*i*_, *β*_*i*_). The S-shaped curve is commonly known as item characteristic curve (ICC) or item response function. In the one-parameter logistic (1PL) model, also referred to as Rasch model, *α*_*i*_ = 1.

DIF attempts to identify those items showing an unexpectedly large difference in item performance between the focal group (households with children) and the reference group (households without children) when the two groups are matched on their total score. In this context, we say the item exhibits DIF when its metric properties differ across groups after the focal and reference groups have been matched on the trait of material deprivation assessed by the questionnaire. Let *Y* denote the response to a particular item on the material deprivation questionnaire. Notice that *Y* is determined solely by the material deprivation intended to be measured by the questionnaire, denoted by *θ*, and the random error. Thus, the probability distribution of *Y* conditional on *θ* is given by *f*(*Y* | *θ*).

For the purpose of this study, we are concerned with comparing the conditional probability of *Y*_*i*_ for the focal (households with children) and reference (households without children) groups. Let *G* denote the grouping variable, with values G = *R* for reference group and *G* = F for focal group. Assuming that error distributions for both groups are identical, if there is no DIF, then logical Eq (1) is satisfied,

$$f\left(Y_{i}\;|\;\theta ,G=R\right)=f\left(Y_{i}\;|\;\theta ,G=F\right).$$ (2)

According to Chalmers [34, p. 117], the “reference group is a baseline group against which all comparisons are to be made, while the focal group is drawn from the population in which DIF is suspected”, providing the rational to our decision of setting *F* as the focal group. For every item, the null hypothesis significance test of no DIF is used.

## Results

Our results presented in Table 3 include likelihood-ratio tests and also AIC, AICc, SABIC, HQ, and BIC statistics for model assessments.

**Table 3:** Likelihood-ratio tests, statistics and p values.

	AIC	AICc	SABIC	HQ	BIC	X2	df	p
I1	-510466	-510433	-502145	-505530	-495789	514466	2	0.000
I2	3423	3457	11744	8360	18100	577	2	0.749
I3	-67939	-67905	-59617	-63002	-53262	71939	2	0.000
I4	-54523	-54490	-46202	-49587	-39846	58523	2	0.000
I5	-62215	-62182	-53894	-57279	-47538	66215	2	0.000
I6	-16119	-16085	-7798	-11182	-1442	20119	2	0.000
I7	-12529	-12495	-4207	-7592	2148	16529	2	0.000
I8	-49302	-49269	-40981	-44366	-34625	53302	2	0.000
I9	-25746	-25712	-17424	-20809	-11069	29746	2	0.000
I10	-8795	-8761	-473	-3858	5882	12795	2	0.002
I11	2932	2965	11253	7868	17609	1068	2	0.586
I12	-2304	-2271	6017	2632	12373	6304	2	0.043

At the level of significance of 5%, the null hypothesis is rejected in 10 out of 12 items, *i*.*e*., it is not rejected in item 2 (p value = 0.749) and item 11 (p value = 0.586). The same is to say that empirical evidence provided by the tests is not strong enough at the 5% level to conclude that, from the perspective of measurement, items related to dimly lit dwelling and pollution, grime or other environmental problems in residential area caused by traffic or industry, perform differently according to the group. All other items seem to perform differently in households with children or without children.

**Fig 1:**
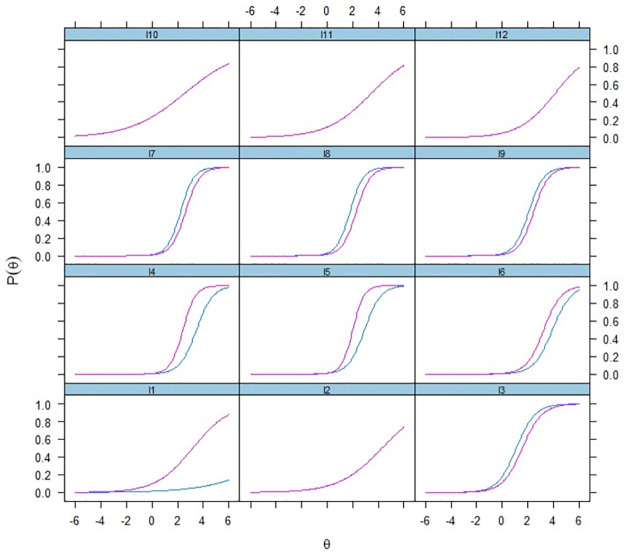
ICC for items of composite index, focal (in blue) and reference (in pink) groups.

The ICC curves presented in Fig 1 illustrate that in some items DIF is in favor of the reference group. In other items, it is in favor of the focal group. In any case, our results show that children poverty assessment or its proxy may be jeopardized by the presence of DIF. We know that children living in households experiencing poverty tend to be “adversely affected by society’s negative perceptions of their families” [35, p. 49]. If, in addition, public policy towards poverty reduction is based on unreliable or invalid indicators, the consequence may be even worse than the lack of state intervention. However, DIF implications in terms of effect size or measurement invariance are beyond the objective of this article.

## Discussion

Our study demonstrated how to statistically check for DIF in the CPSE assessment and has two major implications for policy and further research developments. Although the methodological proposal included in this study is household-based, the composite index of child exposure to material deprivation gives information about children’s living conditions and opportunities that are intrinsically related to children’s wellbeing. Therefore, our approach strives to give important insights for social policy design and monitoring, based on reliable datasets, on an annual basis, and composite indices built upon items which catch the children living conditions. Our paper presents an approach to the analysis of the differential functioning of items and demonstrates its existence in an application to the measurement of CPSE and further develops methodology proposed by [19, 20]. Our findings demonstrate that there is differential item functioning in the assessment of material deprivation in households with or without children. The methodological approach used allows for the interpretation of scale points in terms of what they mean for a child living exposed to poverty and what impact of DIF on scale interpretation is at different scale scores. This is out of the scope of this paper and further research is planned to address invariance measurement related to the presence of DIF.

Furthermore, as [36, p. 16] describe, DIF “is not always a flaw or weakness. Subsets of items that have a specific characteristic in common (*e*.*g*., specific content, task representation) may function differently for different groups of similarly scoring test takers. This indicates a kind of multidimensionality that may be unexpected or may conform to the test framework”, implying the need of addressing the issue with the lens of a multidimensional phenomenon. For example, [37] evaluated DIF in the EPICES questionnaire between different geographical french regions and found several items with DIF in a social deprivation analysis.

The presence of DIF can lead to biased assessments of group differences and confound risk factor and outcomes and therefore have implications for the design, monitoring/evaluation of programs and policies in child poverty. Relying on unreliable or invalid indicators for public policy targeting children’s poverty reduction can have consequences that are potentially worse than having no state intervention at all. If policymakers make decisions and allocate resources based on inaccurate or flawed measurements of poverty, it can lead to ineffective or misguided interventions that fail to address the actual needs of the children. This can result in wasted resources, missed opportunities for effective poverty reduction, and potentially exacerbate the existing social and economic disparities. Therefore, using reliable and valid indicators for guiding public policy to ensure that interventions are targeted and impactful in addressing poverty-related challenges is essential.

In this paper, we utilize DIF hypothesis tests to identify EU-SILC items that exhibit differing performance between households with and without children. This study fills a literature gap by demonstrating statistical specificity in CPSE assessment, which has not been shown in previous research. Additionally, our study contributes by proposing a methodology to enhance the validity and reliability of CPSE assessment using complex sampling survey data. Furthermore, we identify specific items or indicators commonly used for poverty assessment that exhibit DIF, providing valuable insights to the field.
